# 1.3 µm dissipative soliton resonance generation in Bismuth doped fiber laser

**DOI:** 10.1038/s41598-021-85423-y

**Published:** 2021-03-18

**Authors:** H. Ahmad, S. N. Aidit, S. I. Ooi, M. Z. Samion, S. Wang, Y. Wang, J. K. Sahu, A. K. Zamzuri

**Affiliations:** 1 Photonics Research Centre, University of Malaya, 50603 Kuala Lumpur, Malaysia; 2 Physics Department, Faculty of Science, University of Malaya, 50603 Kuala Lumpur, Malaysia; 3 Optoelectronics Research Centre, University of Southampton, Highfield, Southampton, SO17 1BJ UK; 4 Physics Department, Kulliyyah of Science, International Islamic University Malaysia, 25200 Kuantan, Pahang Darul Makmur Malaysia

**Keywords:** Fibre optics and optical communications, Laser material processing

## Abstract

In this work, a Figure-9 (F9) bismuth-doped fiber laser (BiDFL) operating in the dissipative soliton resonance (DSR) regime is presented. The 1338 nm laser used a BiDF as the active gain medium, while a nonlinear amplifying loop mirror (NALM) in an F9 configuration was employed to obtain high energy mode-locked pulses. The wave breaking-free rectangular pulse widened significantly in the time domain with the increase of the pump power while maintaining an almost constant peak power of 0.6 W. At the maximum pump power, the mode-locked laser delivered a rectangular-shaped pulse with a duration of 48 ns, repetition rate of 362 kHz and a radio-frequency signal-to-noise ratio of more than 60 dB. The maximum output power was recorded at around 11 mW with a corresponding pulse energy of 30 nJ. This is, to the best of the author’s knowledge, the highest mode-locked pulse energy obtained at 1.3 μm as well as the demonstration of an NALM BiDFL in a F9 configuration.

## Introduction

Passively mode-locked fiber lasers have attracted significant interest amongst researchers worldwide due to their significant potential for use in a wide variety of applications such as micromachining^1^, biomedical diagnosis^2,3^, supercontinuum generation^4^ and molecular spectroscopy^5,6^. Unlike their bulk optic counterparts, mode-locked fiber lasers in particular have the additional advantages of being robust and compact ultrashort pulse sources while having excellent beam qualities. Furthermore, by managing the various parameters of the laser cavity, the mode-locked pulses can be made to operate in different regimes, including the conventional soliton pulse, stretched pulse and the self-similar pulse.

Recently, there has been an increased effort to generate high energy, rectangular-shaped mode-locked pulses for the use in all-optical square-wave clocks^7^, laser micromachining^8^ and optical sensing^9^ applications. These rectangular pulses are normally obtained from a mode-locked fiber laser operating in the dissipative soliton resonance (DSR) regime. These type of pulses were initially obtained in the frame of the cubic-quintic complex Ginzburg–Landau equation^10^, where the width of the mode-locked pulse in the DSR regime increases with the pump power without pulse breaking, while simultaneously keeping the peak power at a constant level. As such, the pulse energy could be theoretically increased indefinitely compared to the conventional soliton pulse, stretched pulse and self-similar pulse, where the energy is typically limited to 0.1–13.8 nJ^11–16^. Furthermore, DSR pulses exhibit a unique compression dynamic that is different from other soliton pulses^17^. The detailed evolution of the DSR pulse compression has been numerically and experimentally demonstrated by Li et al*.* in 2016. The DSR pulse was compressed down to 760 fs from an original pulse width of 63 ps using a grating pair^18^. This approach spurred significant research efforts on high energy rectangular-shape mode-locked pulses from many different types of fiber laser configurations with cavity parameters favoring the DSR regime. However, while DSR mode-locked operation has since been investigated extensively at various wavelength regimes using erbium-^19–24^, erbium:ytterbium-^25–32^ and thulium-doped fiber^33–40^ laser cavities, there has been little to no progress made in the 1.3 μm region.

In recent years, the availability of rare-earth doped fluoride fiber has spurred the development of fiber lasers operating at 1.3 μm. This is mainly from the emission band of Pr^3+^ cations in fluoride fiber pumped by commercial 1.02 μm laser diode^41–45^. Nevertheless, the gain of Pr^3+^-doped fluoride fiber amplifier is hindered by a poor quantum efficiency due to the high rate of multi-phonon relaxation process, thus limiting the fiber laser efficiency. Additionally, the feasibility of a compact all-fiber laser in 1.3 μm was also restricted due to the number of complexities that need to be settled such as splicing fluoride fibers to standard telecom fiber (SMF-28). Of late, the advancement of bismuth (Bi)-doped active fibers has shown significant progress in developing amplifiers and lasers. Efficient amplifiers and CW lasers based on bismuth-doped fibers (BiDF) in different glass hosts i.e. aluminosilicate, phosphosilicate and germanosilicate were demonstrated. In particular, bismuth-doped phosphosilicate fibers have been vigorously studied due to its ability to efficiently amplify an optical signal in the second telecommunication window from 1300 to 1360 nm^46–48^ as well as its compatibility with SMF-28 fiber for low loss splicing. The first bismuth-doped phosphosilicate fiber amplifier operating in the range 1300–1340 nm was realized in 2009 with a maximum gain of ~ 32 dB at 1320 nm^49^. Thus far, several studies have demonstrated the use of bismuth-doped phosphosilicate fiber as an active medium for 1.3 μm pulsed fiber laser. For instance, in 2013, Gumenyuk et al*.* obtained mode-locked pulses in a bismuth-doped phosphosilicate fiber laser using a SESAM^50^. Khegai et al*.* further reported a 1.3 μm picosecond mode-locked pulse with an energy of 1.65 nJ in a figure-of-8 bismuth-doped phosphosilicate fiber laser^51^. The pulse energy was increased to 8.3 nJ with a BiDF amplifier. Later, in 2017, Thipparapu et al*.* observed a stable 1340 nm mode-locked pulse in a simple ring bismuth-doped phosphosilicate fiber laser cavity without any saturable absorber^52^. The pulse was further amplified using a master oscillator power amplifier (MOPA) and a maximum single pulse energy of 2.9 nJ was achieved. Recently, a 1.3 μm picosecond mode-locked pulse fiber laser was successfully demonstrated by Khegai et al*.* with single-walled carbon nanotubes (SWCNTs)^53^. Besides, femtosecond pulses in all-fiber scheme have also been reported at this wavelength region utilizing a relatively large Raman shift of phosphosilicate fiber^54,55^ and self-phase modulation (SPM)^56^. Qin et al*.* demonstrated watt-level synchronously pumped all-fiber optical parametric chirped-pulse amplifier at 1.3 μm region with a compressible pulse, down to ~ 306 fs^57^. Although some research has been carried out on mode-locked pulse-based bismuth-doped fiber lasers (BiDFLs), limited efforts have been made to investigate DSR generation. Among the first few demonstrations of DSR BiDFL was reported by Zhao et al*.* in 2017 operating at 1169.5 nm^58^. The DSR laser which was based on the nonlinear polarization rotation (NPR) technique yielded a maximum pulse energy of ~ 24.8 nJ. However, the DSR BiDFL operating in the 1.3 μm region has not been investigated.

In this work, the generation of high energy mode-locked pulses in a figure-of-9 (F9) BiDFL operating at 1338 nm is reported. An NALM was employed as the mode-locking element. The width and energy of the rectangular-shaped mode-locked pulse were observed to increase linearly with the pump power, reaching a maximum of 48 ns and 30 nJ respectively. No pulse breaking was observed. The experimental results match well with the previous works, verifying that the laser works in the DSR regime. These are, to the best of our knowledge, the highest energy mode-locked pulse observed in a BiDFL operating at the 1.3 µm region.

## Experimental setup

**Figure 1:**
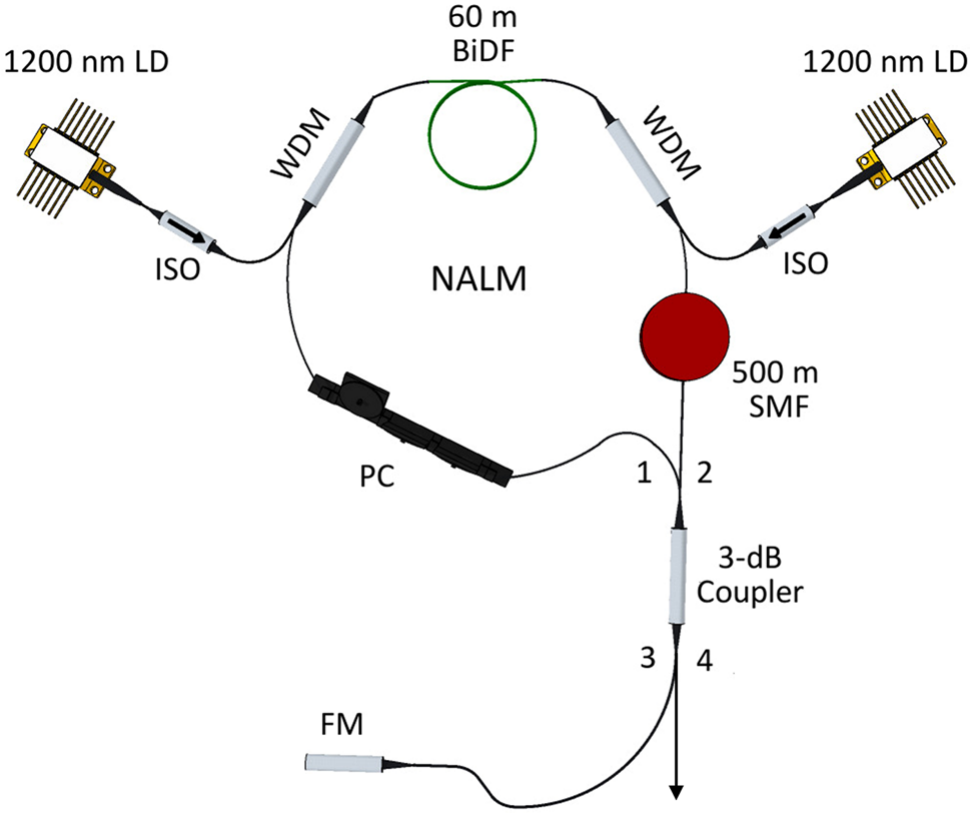
Schematic diagram of the DSR F9 BiDFL. This figure was drawn using SketchUp Make 2017 (Basic), Software Version: Windows 64-bit 17.2.2555, available at https://www.sketchup.com/download/all.

The configuration for the DSR BiDFL based on the F9 is depicted in Fig. 1. The laser cavity consisted of a bi-directional loop in which amplification is provided by the gain medium. The gain medium was a 60-m long BiDF, pumped by two laser diodes at a wavelength of 1200 nm. Each of the 1200 nm pump was protected by a 1200 nm isolator (ISO) from back reflections. The BiDF had core and cladding diameters of 9 and 125 μm, respectively. It has a group velocity dispersion (GVD) of approximately − 0.3 ps^2^/km at 1338 nm^53^. A 500-m long SMF was placed in the bi-directional loop as to increase the nonlinearity. The GVD parameter of the SMF is − 2.25 ps^2^/km at 1338 nm. A polarization controller (PC) was used to adjust the intra-cavity polarization states as to obtain an optimized output of the mode-locked pulse. The bi-directional loop was completed at the port 1 and 2 of a 3-dB coupler, giving a total length of about 571 m. A 1310 nm Faraday mirror (FM) was connected to port 3 of the coupler, while port 4 was taken as the output. The FM would reflect majority of the signal back into the main loop, providing feedback to the laser resonator. The net cavity dispersion was calculated to be − 1.17 ps^2^.

The idea of mode-locking operation in the F9 laser configuration is similar to that of mode-locked in figure-of-8 (F8) configuration with a NALM. In an NALM F8 laser configuration, pulse formation depends on the difference of nonlinear phase shift accumulated between the clockwise and counterclockwise propagating light in the gain loop (NALM loop). The signal was subsequently transmitted through the 3 dB coupler once the asymmetric nonlinear phase shift reached a certain level. Typically, the two output ports of the 3 dB coupler (port 3 and 4 in Fig. 1) are connected together with an isolating element and an additional tapped coupler, forming a unidirectional loop. However, this passive unidirectional loop was not required in the F9 laser configuration, which simplifies the laser construction. The unidirectional functionality in the F9 was achieved by reflecting the pulse that exits the NALM loop through the FM, ensuring feedback to the laser resonator.

## Results and discussion

**Figure 2:**
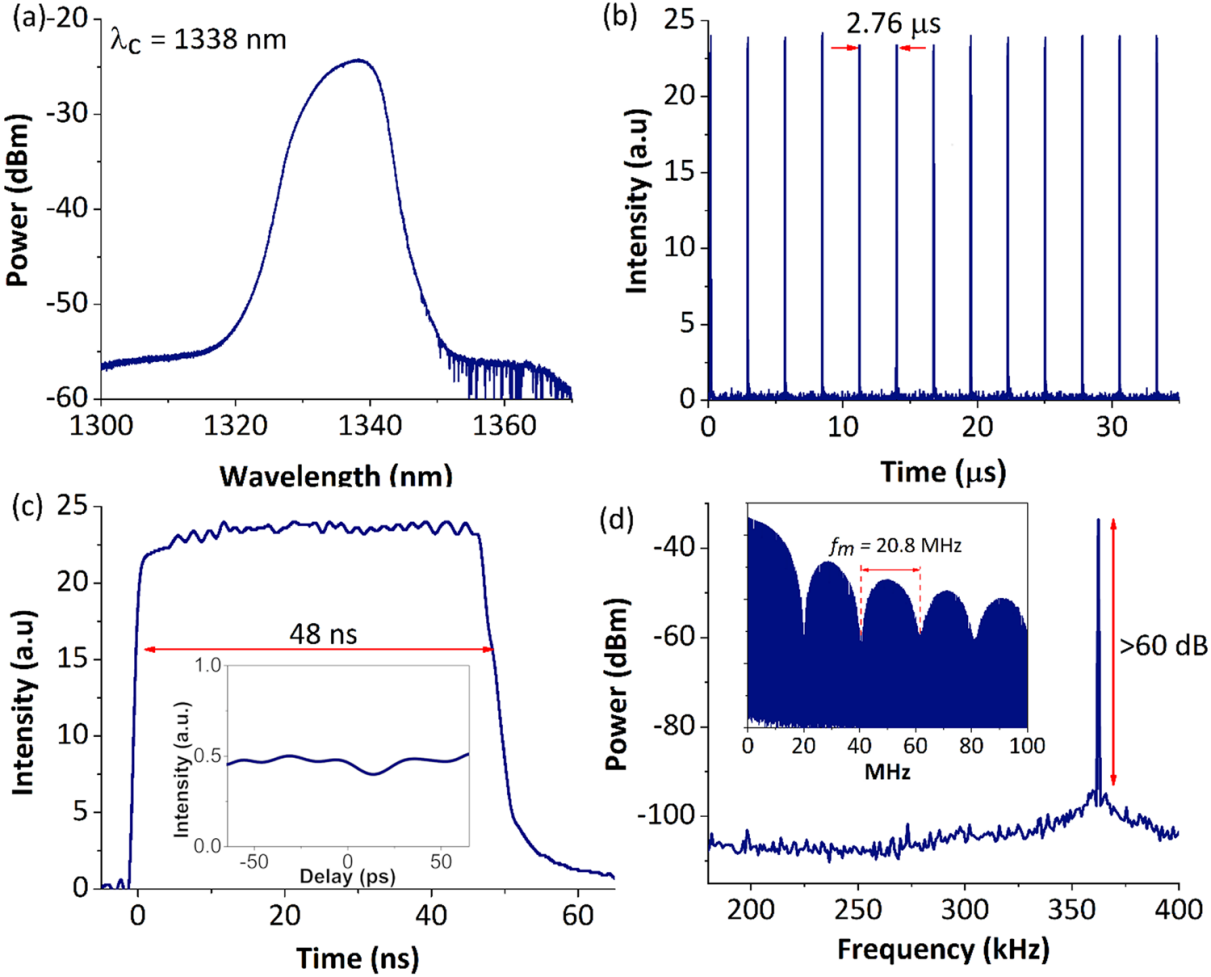
DSR laser emission at pump power of 1.04 W. **(a)** Optical spectrum, **(b)** pulse train, **(c)** temporal profile of single square pulse (inset: autocorrelation trace) and **(d)** RF spectrum at fundamental frequency (inset: RF spectrum over a wide span of 100 MHz).

A stable mode-locking operation could be observed with a proper adjustment of the intra-cavity polarization state using the PC. Once the proper position of the PC was found and fixed, a mode-locking operation was obtained at a pump power of 660 mW. The output characteristics of the mode-locking at pump power of 1040 mW are illustrated in Fig. 2, where Fig. 2a shows the mode-locked optical spectrum at a resolution of 0.02 nm. The optical spectrum has a central wavelength of 1338 nm and a 3-dB bandwidth of 10 nm. Figure 2b shows the oscilloscope trace of the mode-locked pulse train with a pulse repetition rate of 362 kHz. The pulse train was observed with a uniform pulse interval of 2.76 μs that coincides well with the cavity round-trip time and therefore confirming the fundamental operation of the mode-locking. Figure 2c shows the single pulse profile of the mode-locking. The mode-locked laser emits a flat-top rectangular pulse with a duration of 48 ns. The flat-top rectangular temporal profile is a typical feature of mode-locked pulses operating in the DSR regime. Note that a mode-locked laser operating in the noise-like pulse (NLP) regime could also generate a flat-top rectangular pulse, whose pulse dynamics are very similar to that of DSR pulse. The difference between them is that the DSR pulse is a single pulse, whereas the NLP is a wave packet consisting of many sub-pulses in sub-picoseconds with varying width and peak intensities. Thus, the autocorrelation (AC) trace of an NLP usually shows a sub-picosecond coherent spike on top of a wide pedestal, which is a typical characteristic of an NLP^59,60^. As such, in order to verify the operation regime of the generated pulse, the autocorrelation (AC) measurement was performed. The scan range was set to a maximum of 150 ps, which was sufficient to detect any sub-picosecond coherent spike. The autocorrelation (AC) trace of the rectangular wave is shown in the inset of Fig. 2c. No NLP-characteristic fine structure or spike was observed, on top of noise level. This verifies that the laser was operating in the DSR regime^20,36, 61^. Furthermore, the DSR pulse has a regular phase inside it^17^ that allows it to be compressed^18^. This could be achieved by the dispersive Fourier transform (DFT) technique^62^, which will be of interest in future studies. Additionally, as seen from Fig. 2d, the output RF spectrum which was recorded with a 100 Hz resolution bandwidth has a peak at the fundamental frequency of 362 kHz with signal-to-noise ratio (SNR) of > 60 dB. Another intrinsic feature of DSR mode-locked pulse is the characteristic of an RF spectral modulation, which can be observed from the inset of Fig. 2d. A large RF spectral modulation, *f*_*m*_ of 20.8 MHz can be seen from the spectrum, originating from the width of the emitted pulse, *τ* which was 48 ns. The RF spectral modulation satisfies a reciprocal relation of *f*_*m*_ = 1/*τ*, and it changes uniformly with pump power^63^.

**Figure 3:**
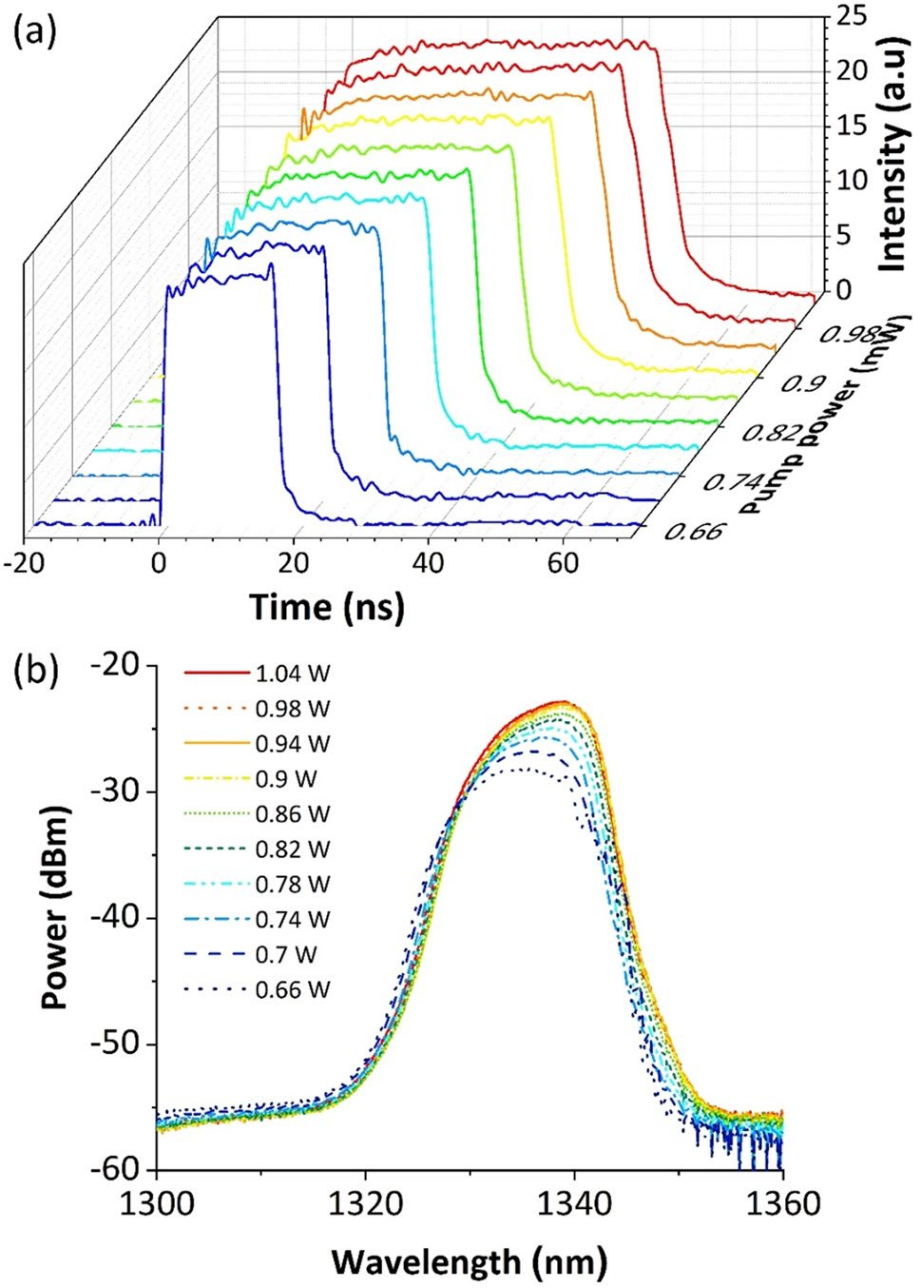
**(a)** Single-pulse envelope as the pump power increases and **(b)** the corresponding optical spectrum.

The DSR features were further investigated at a fixed polarization state. Figure 3a shows the single pulse evolution with the pump power. It is apparent that when the pump power increases, the pulse kept its rectangular temporal profile and widens significantly in the time domain. Meanwhile, the pulse peak power was clamped, which is a typical feature of a mode-locking operation in the DSR regime. On top of this, there was no multi-pulse operation or pulse breaking observed throughout the temporal broadening process. This indicates a stable operation of the mode-locked laser. The corresponding optical spectrum is depicted in Fig. 3b. One can clearly see that the spectrum was slightly shifted to a longer wavelength and increased in its intensity as the pump power was increased. The newly generated energy was accumulated at the center part of the spectrum due to the peak-power-clamping effect ^64^. Normally, when the pulse peak power was clamped to a low value, the accumulation of the nonlinear phase shift due to the self-phase modulation would also be reduced, thereby reducing the spectral broadening width. The optical spectrum would thus be confined within the cavity filter transmission window, circumventing pulse breaking. When the pulse breaking is circumvented, the pulse width increases with the increment of pump power to store higher energy.

**Figure 4:**
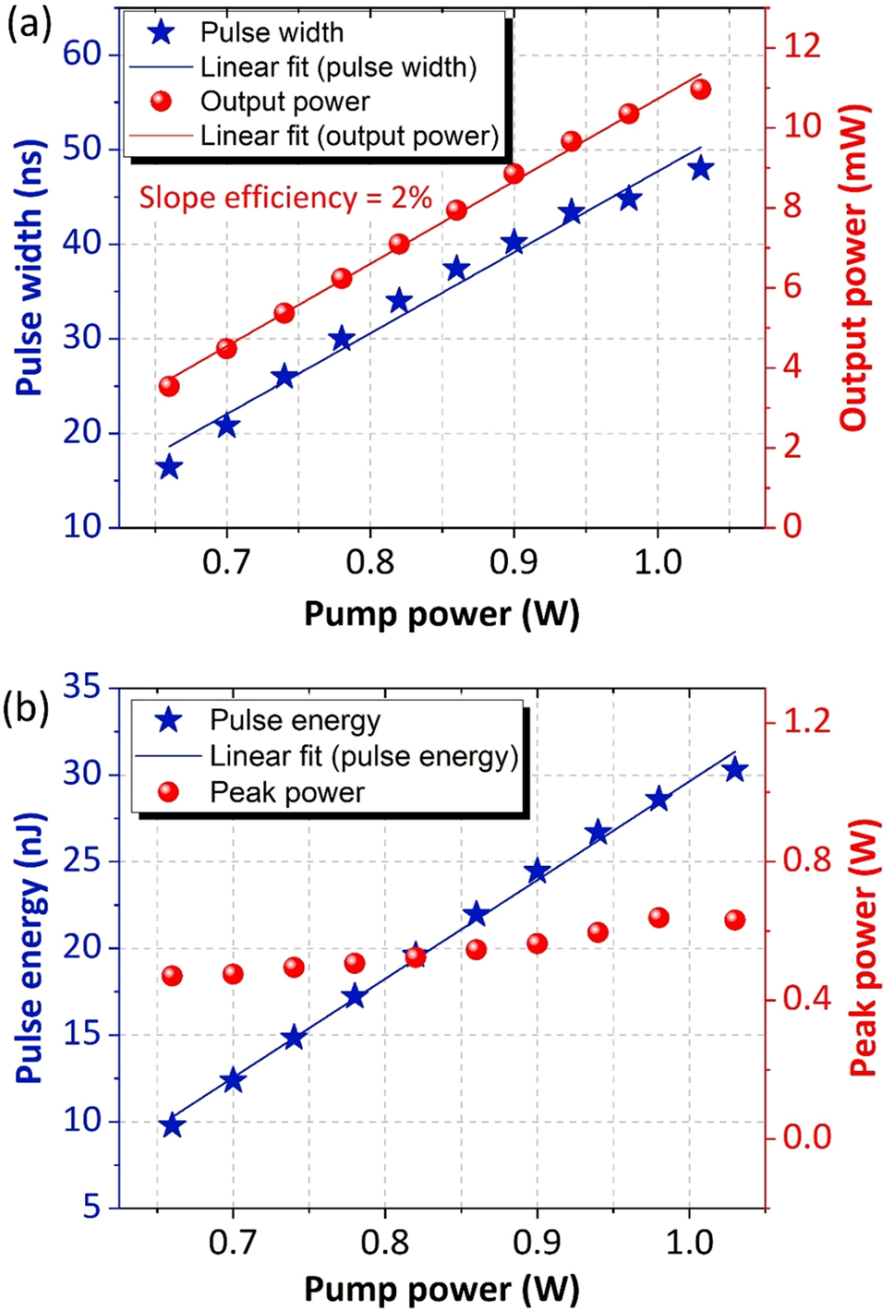
**(a)** Single-pulse envelope as the pump power increases and **(b)** the corresponding optical spectrum.

To better understand the characteristic of the mode-locked pulse in the DSR regime, the variation of pulse width, output power, pulse energy and peak power against the pump power are plotted in Fig. 4. As seen here, the output power increases linearly with the pump power. The maximum average output power was 11 mW recorded at the pump power of 1040 mW. The calculated slope efficiency of the laser is about 2%. The pulse width was also observed to increase with the pump power. As the pump power increases from 660 to 1040 mW, the mode-locked pulse experiences a linear broadening in time domain, from 16.4 ns to 48 ns. The corresponding pulse energy and peak power are calculated and plotted in Fig. 4b. Unlike the conventional soliton, where the single pulse energy was limited by the soliton area theorem, the single pulse energy of mode-locked pulse operating in the DSR regime can generate an infinitely large power without pulse breaking. This is attributed to the strong peak-power-clamping effect induced by the long length of NALM^64^.

In optical fiber cavities with a strong peak-power-clamping effect, the pulse peak power will be clamped at a low value and therefore prevent the pulse breaking effect. Correspondingly, the pulse energy could be increased to a large value. As predicted, the pulse energy is proportional to the pump power, reaching to a maximum of 30 nJ at a pump power of 1040 mW. The maximum pulse energy was limited by the availability of the pump power. Simultaneously, the peak power remained at a comparable level and reached a maximum of 0.6 W. Generally, these observations are in accordance with previous studies indicating that the mode-locked laser was operating in the DSR regime^10,65,66^.

**Figure 5:**
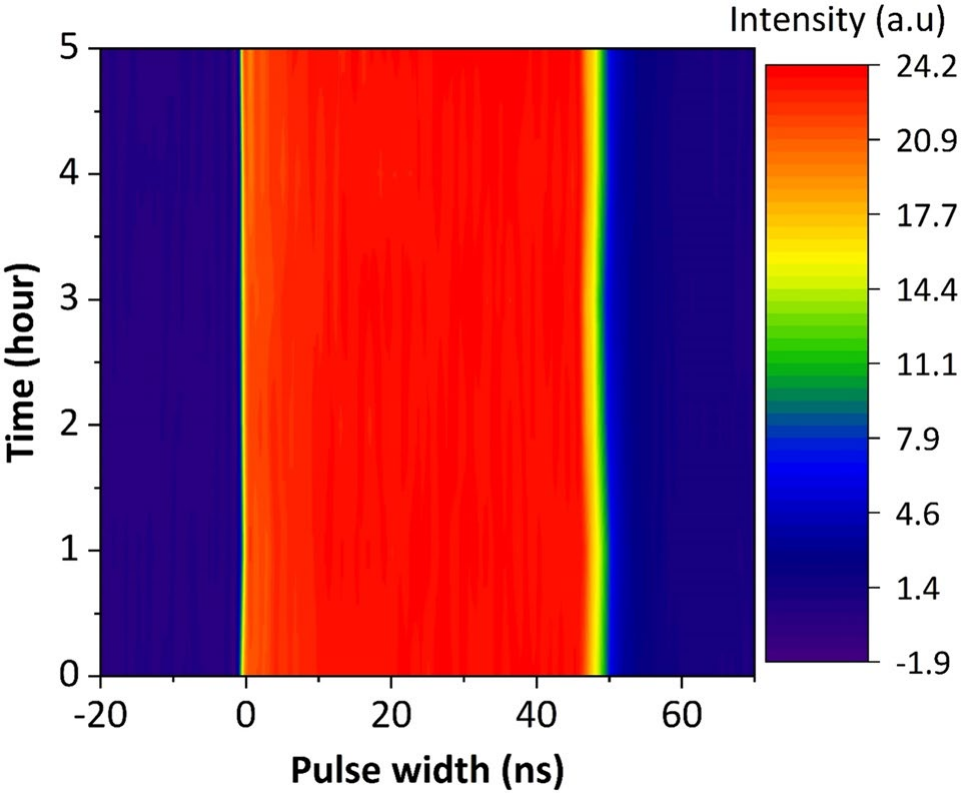
Stability measurement of the DSR operation made by repeateadly scanning the pulse profile during a ~ 5-h test.

**Figure 6:**
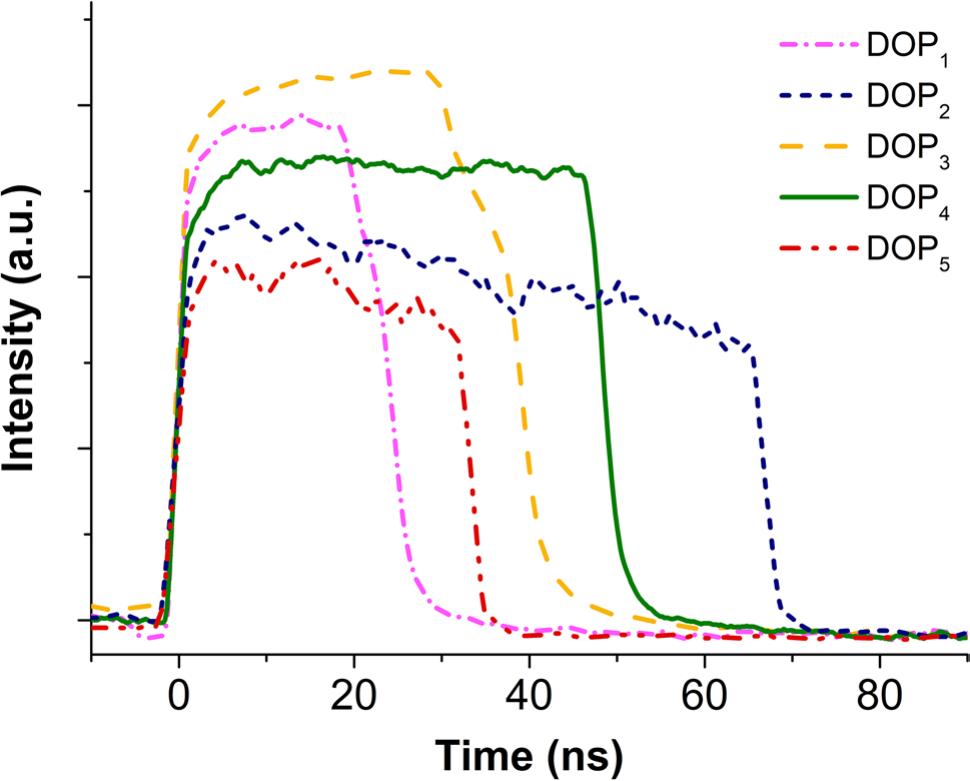
Profiles of five square pulses at different degree of polarizations (DOPs).

Additionally, the stability of the mode-locked laser was measured by oscilloscope traces taken over a period of 5 hours at 1 hour intervals at a pump power of 1040 mW. As plotted in Fig. 5, there was no significant change in the pulse width as well as the output power over the entire test period. This indicates excellent repeatability and long-term stability of the high-energy mode-locked laser. Further adjustment of the PC enables multiple stable state of DSR operation, which can be seen in Fig. 6. A series of stable square pulses with different pulse width and peak power was observed at different degree of polarization (DOP). Table 1 summarizes the pulse width and peak power of square pulses at different DOP. It can be seen that the square pulse exhibits broadest width of 67 ns at DOP of 26.7% with corresponding peak power calculated to be around 198 mW whereas the highest peak power of 600 mW was recorded when the DOP was set at 31.8%. The observed multistability of DSR operation could be attributed to the variation of NALM transmission with DOP. Since the transmittance of NALM depends on the difference of the accumulated nonlinear phase shift of counter propagating beam, change in the polarization state will affect the overall transmittance. Particularly, when the PC is adjusted, the overall NALM transmittance changed, which subsequently altered the threshold peak power of the peak-power-clamping effect and amplitude of the square pulse^67^. This resulted in a variety of square pulses with different peak powers and pulse widths. The laser output was analyzed with the help of the Rohde & Schwarz 1 GHz oscilloscope and the PAX1000 inline polarimeter.

**Table 1:** Multiple stable state DSR operation at different DOPs.

DOP	DOP (%)	Pulse width (ns)	Peak power (mW)
1	13.4	24.6	504
2	26.7	67	198
3	30.2	39	380
4	31.8	48	600
5	33	33.4	354

In our previous work focusing on high energy mode-locked laser in 1.3 μm^68^, a Pr^3+^-doped fluoride fiber was utilized as the amplifying medium and a typical figure-of-8 configuration with a nonlinear optical loop mirror (NOLM) served as the mode-locking mechanism. This approach yielded DSR mode-locked pulse with an energy of 2.3 nJ and a poor slope efficiency of 0.2%. In contrast, the present work using the F9 configuration yields a much higher energy by a factor of 13 and a better slope efficiency of 2% as compared to previous works. The enhanced pulse energy and slope efficiency in this work could be attributed to the F9 configuration, as well as the use of suitable gain medium for 1.3 μm signal amplification.

## Methods

### Laser characterization

The output of the laser was analyzed using a Yokogawa AQ6375 optical spectrum analyzer, a Thorlabs S148C power meter, APE pulseCheck 150 USB autocorrelator and using a Thorlabs DET01CFC InGaAs photodetector before connecting it to a Rohde & Schwarz 1 GHz oscilloscope and an Anritsu MS2683A radio frequency spectrum analyzer (RFSA).

## Conclusion

In summary, we have demonstrated to the best of author’s knowledge, the first mode-locked pulse generation in a DSR F9 BiDFL operating at 1.3 μm wavelength region. The wave-breaking free rectangular shaped pulse broadens from 16.4 to 48 ns with an increase of pump power, simultaneously maintaining an almost equal peak power of 0.6 W. At the maximum pump power, the generated pulses have an average output power of 11 mW with a repetition rate of 362 kHz and pulse width of 48 ns, as well as a pulse energy of 30 nJ. This work demonstrated the potential of the BiDF operating under the DSR regime at 1.3 μm.
